# Editorial: Challenges in language evolution research

**DOI:** 10.3389/fpsyg.2023.1233239

**Published:** 2023-09-27

**Authors:** Ana Mineiro

**Affiliations:** ^1^Faculty of Health Sciences and Nursing, Universidade Católica Portuguesa, Porto, Portugal; ^2^Centre for Interdisciplinary Research in Health (CIIS), Universidade Católica Portuguesa, Porto, Portugal

**Keywords:** human tongue, language, evolution, teaching, pantomime

The scope of the Research Topic was to bring together the contributions of researchers from different backgrounds in the field of language origins.

The evolution of human language has been discussed for centuries from different perspectives. Proposals that the semiotic system of gesture played a pivotal role in the evolution of language have been, and continue to be, influential (Zlatev, 2008; Zywiczyński et al., 2018; Zlatev et al., 2020). This statement, however, illustrates not so much a specific theory but an axis of debate in language origins, along which “gesture-first” proposals (Corballis, 2003, 2009; Arbib, 2012; Mineiro et al., 2017, 2021) traditionally compete with “speech-first” approaches (Bickerton, 1990).

One perspective within our Research Topic of articles is the crucial role of the emergence of the human-specific tongue. Ekström and Edlund, in their article entitled *Evolution of the human tongue and emergence of speech biomechanics*, provide a view into the cruciality of the emergence of the human-specific tongue to the evolution of human articulate speech. They state that the tongue properties and morphology were a turning point in the evolution of human articulate speech. It provided the possibility of mapping articulatory targets via the exaptation of manual-gestural mapping capacities evident in extant great apes.

Another view, by Ferretti in an article within this Research Topic entitled, *On the Influence of Thought on Language: A Naturalistic Framework for the Pantomimic Origins of Human Communication*, emphasizes pantomime as an ideal expressive means for bootstrapping the evolutionary foundations of language origins. Pantomime can be angled as a privileged lens for investigating language origin and evolution, partly due to its motivated iconic character of pantomime while compared with the arbitrary and abstract features of language and partly due to the way it obliges rethinking the relationship between thought and language.

Another research vision is rooted in the evolution of teaching as one of the main factors that lead to increasingly complex communicative systems in the hominin species. Gärdenfors, in a paper entitled *Teaching as an evolutionary precursor to language* defends that earlier analyses of the evolution of teaching demonstrate that pantomime seems to be the earliest evolutionary unique human capacity and that could also be explained via the evolution of a theory of mind.

A totally different perspective given teaching comes with Alhadi Ali Ahmed et al. in their article entitled *An in-depth analysis of the representation of speech acts and language functions in Libyan public high school English Textbooks* stating that pragmatic potential competence is the turning point to use speech acts and language functions.

The selection of papers within this Research Topic provides a picture of some of the current challenges in language evolutionary research.

## Author contributions

The author confirms being the sole contributor of this work and has approved it for publication.
